# A computational study of the structure and function of human Zrt and Irt-like proteins metal transporters: An elevator-type transport mechanism predicted by AlphaFold2

**DOI:** 10.3389/fchem.2022.1004815

**Published:** 2022-09-20

**Authors:** Andrea Pasquadibisceglie, Adriana Leccese, Fabio Polticelli

**Affiliations:** ^1^ Department of Sciences, Roma Tre University, Rome, Italy; ^2^ National Institute of Nuclear Physics, Roma Tre Section, Rome, Italy

**Keywords:** ZIP, metal transporters, protein structure prediction, elevator-type mechanism, binuclear metal center

## Abstract

The ZIP (Zrt and Irt-like proteins) protein family includes transporters responsible for the translocation of zinc and other transition metals, such as iron and cadmium, between the extracellular space (or the lumen of organelles) and the cytoplasm. This protein family is present at all the phylogenetic levels, including bacteria, fungi, plants, insects, and mammals. ZIP proteins are responsible for the homeostasis of metals essential for the cell physiology. The human ZIP family consists of fourteen members (hZIP1-hZIP14), divided into four subfamilies: LIV-1, containing nine hZIPs, the subfamily I, with only one member, the subfamily II, which includes three members and the subfamily gufA, which has only one member. Apart from the extracellular domain, typical of the LIV-1 subfamily, the highly conserved transmembrane domain, containing the binuclear metal center (BMC), and the histidine-rich intracellular loop are the common features characterizing the ZIP family. Here is presented a computational study of the structure and function of human ZIP family members. Multiple sequence alignment and structural models were obtained for the 14 hZIP members. Moreover, a full-length three-dimensional model of the hZIP4-homodimer complex was also produced. Different conformations of the representative hZIP transporters were obtained through a modified version of the AlphaFold2 algorithm. The inward and outward-facing conformations obtained suggest that the hZIP proteins function with an “elevator-type” mechanism.

## 1 Introduction

Along with iron, copper, manganese, cobalt, molybdenum, and others, zinc is a member of the transition metal series of the periodic table of elements. These trace elements are involved in a plethora of biological functions including oxygen transfer, enzyme catalysis, protein structural organization, and cellular regulation (Maret, 2013). As a result, imbalances in the concentration of zinc and other metals in the cell can lead to dysfunction and disease (Bleackley and MacGillivray, 2011). For example, zinc deficiency can cause immunodeficiency and alter brain development and function (Hambidge, 2000), while its excess can cause cellular toxicity (Chasapis et al., 2020). Therefore, intracellular zinc concentration is constantly regulated by zinc transporters (Kambe et al., 2021). Among the known metal transporters, members of the SLC39 (Zrt-, Irt-like Proteins or ZIP) family are membrane proteins responsible for the transport of metal ions, including mainly zinc, from the extracellular space, or from intracellular organelles, to the cytoplasm (Kambe et al., 2021). This family is particularly important because its members are involved in zinc uptake and homeostasis of at least three essential metal ions: zinc, iron, and manganese (Hu, 2021). Mutations in ZIP transporters are known to be associated with several human diseases. Loss-of-function mutations in the SLC39A4 gene, which codes for the ZIP4 transporter, cause acrodermatitis enteropathica (AE) (Küry et al., 2002). Recessive mutations in the SLC39A13 gene, which codes for the ZIP13 transporter, cause a subtype of Ehelers-Danlos syndrome (EDS) (Jeong et al., 2012). Zinc deficiency is also known to cause alterations in the immune system, abnormalities in T cells, natural killer cells, and monocytes, and results in reduced antibody formation (Hambidge, 2000). Further, changes in intracellular zinc levels, due to altered expression of ZIP family transporters, are associated with tumor growth and progression, and metastasis formation (Hu, 2021).

The ZIP transporter family includes tens of thousands of both eukaryotic and prokaryotic members that have been identified at all phylogenetic levels (Hu, 2021). Fourteen human ZIPs (hZIP1-14) have been identified in the human genome, with different tissue distribution and physiological function. The ZIP family can be divided into four subfamilies, distinguished on the basis of the degree of conservation of gene sequences (Gaither and Eide, 2001). The fourteen hZIPs are distributed among the four subfamilies as follows: hZIP9 belongs to subfamily I; hZIP1, hZIP2 and hZIP3 belong to subfamily II; hZIP11 belongs to the subfamily GufA; hZIP4, hZIP5, hZIP6, hZIP7, hZIP8, hZIP10, hZIP12, hZIP13, and hZIP14 belong to the LIV-1 subfamily, also called LZT (LIV1-subfamily of ZIP zinc Transporters). The subfamily LIV-1 is in turn dived in four subgroups (Zhang et al., 2016).

From a structural viewpoint, the available literature converges in indicating the presence in most ZIP family members of eight transmembrane α-helices (TMs), having the N- and C-terminal ends facing the extracellular space, or the vesicular lumen for intracellular ZIPs (Hu, 2021). This topology was confirmed by the crystallographic structure of the transmembrane domain (TMD) of the bacterial *Bordetella bronchiseptica* ZIP (BbZIP) (PDB ID: 5TSA; Zhang et al., 2017). Two accessory domains have also been identified that appear to play regulatory roles in the transport function of the protein: the extracellular N-terminal domain (Extracellular domain or ECD) and a long intracellular histidine rich loop (Intracellular loop or IL2) between TM3 and TM4 (Zhang et al., 2016; Zhang et al., 2020a.). The ECD is an accessory domain that is not absolutely necessary for metal transport; in fact, the existence of a complete ECD has only been observed in vertebrates (Zhang et al., 2016). Even in the hZIP family, this subdomain is highly variable and not always present. The only structural information available for mammalian ZIPs is the crystal structure of the *Pteropus alecto* ZIP4 ECD (PaZIP4-ECD) (PDB ID: 4 × 82; Zhang et al., 2016). However, the ECD plays crucial auxiliary roles, as in the case of ZIP4, where it was observed that deletion of the ECD (ZIP4-∆ECD) does not alter the folding of the protein, nor its expression levels on the membrane, but causes a 70–80% reduction in V_max_ in zinc transport (Zhang et al., 2016). Furthermore, seven of the 15 missense mutations responsible for AE genetic disease are located in the ECD of ZIP4 and mutations in ZIP4-ECD affect the metal transport (Küry et al., 2002; Zhang et al., 2016). PaZIP4 ECD displays high sequence similarity with hZIP4-ECD (68% sequence identity). PaZIP4 ECD displays a homodimeric structure in which each monomer shows two independent subdomains, the N-terminal histidine rich domain (HRD), mainly involved in ion transport, and the C-terminal PAL containing domain (PCD), fundamental for dimerization (Zhang et al., 2016).

Regarding IL2, as this is a highly disordered domain, through NMR studies it has been observed to be consistent with a random coil with minor propensities for helices and β-strands in regions implicated in post-translational modifications (Bafaro et al., 2019).

The crystallographic structure of BbZIP shows that the 8 TMs form a helix bundle composed of two cylinders: TM2, TM4, TM5, and TM7 form the inner cylinder and the transport channel, while TM1, TM3, TM6, and TM8 form an outer cylinder in contact with membrane lipids (Supplementary Figure S1). The same structure revealed the presence of two metal binding sites identified as M1 and M2, which constitute a binuclear metal center (BMC). The metal transport channel can be identified in the crystal structure of BbZIP in which S106 appears to be a gating residue at the entrance of the pore in the crystallized inward-facing conformation (Zhang et al., 2017). Between S106 and the BMC, several hydrophobic residues form a hydrophobic core, occluding the pore and preventing metal ions from entering the transporter channel (Zhang et al., 2017). In order for the substrate to pass through this first hydrophobic region and reach the BMC, a conformational change from an inward-facing to an outward-facing state is required. The conformational change of BbZIP from the inward-facing to the outward-facing conformation was simulated by molecular dynamics (MD) allowing to identify the putative residues involved in metal binding (Sharma and Merz, 2021). At the level of the metal center are residues that have a good tendency in coordinating transition metals, which are conserved in most ZIPs (Hu, 2021). According to Zhang and others, the six potential residues involved in metal ion coordination at the metal center level are exclusively present within the “P1-P2-x-x-P3” motif on TM4 and TM5. The positions indicated by x are generally small or hydrophobic residues found at the interface between the two helices. Residues at position P1 on TM4 (TM4-1) and TM5 (TM5-1) coordinate the metal in M1. Residues in position P2 on TM4 (TM4-2) and TM5 (TM5-2) coordinate the metal in M2. Residues at position P3 on TM4 (TM4-3) and TM5 (TM5-3) can coordinate metal in both M1 and M2 (Zhang et al., 2020b). The crystal structure of BbZIP allowed also to identify residues that appear to be involved in the metal exit pathway to the cytoplasm (Zhang et al., 2017).

Moreover, through mutagenesis studies and transport assays conducted on hZIP4, M1 was shown to be the primary transport site, which is absolutely necessary for transport, while M2 appears to have an auxiliary role presumably by acting as an additional transport site that can modulate the properties of the primary transport site (Zhang T. et al., 2020). Indeed, in some ZIPs, a lysine residue appears to replace a residue involved in metal coordination at the M2 site, which would prevent by electrostatic repulsion the binding of the second metal ion to the M2 site. According to a study conducted on hZIP2, the lysine residue occupying M2 is critical for metal transport activity (Gyimesi et al., 2019). It was observed by prediction of the pKa values of the residues critical for transport, that the presence of the lysine residue (K203 in hZIP2) at M2 affects the pKa of the neighboring aspartate (D482 in hZIP2) that binds the metal ion at M1 (Gyimesi et al., 2019).

Scarce structural data on hZIPs and poor knowledge of the possible transport mechanism prompted us to carry out an extensive *in silico* study of the structure, metal coordination and conformational landscape properties, predicted through an *ad hoc* implementation of Alphafold2, of each member of the human ZIP family. Results obtained shed new light on the molecular details of the hZIP family members and allow to hypothesize an “elevator-type” mechanism for ion translocation.

## 2 Computational methods

### 2.1 Sequences retrieval, analyses and alignment

The full-length protein sequences, for each hZIP, were retrieved from the UniProt webserver (The UniProt Consortium, 2021) (Supplementary Materials Table 1). For each hZIP, the presence of a probable signal peptide was predicted using SignalP6.0 web-server (https://services.healthtech.dtu.dk/service.php?SignalP-6.0; Teufel et al., 2022). The multiple sequence alignment (MSA) between the fourteen hZIP mature sequences (without the predicted signal peptide) and the BbZIP sequence (PDB ID: 5TSA; Zhang et al., 2017) were produced with the MUSCLE algorithm (Edgar, 2004) implemented in Jalview (Waterhouse et al., 2009), with default settings.

**TABLE 1:** Predicted signal peptide for each hZIP.

Protein	Signal peptide residues
hZIP1	Absent
hZIP2	Absent
hZIP3	Absent
hZIP4	1-22
hZIP5	1-24
hZIP6	1-28
hZIP7	1-27
hZIP8	1-22
hZIP9	Absent
hZIP10	1-25
hZIP11	Absent
hZIP12	1-23
hZIP13	1-32
hZIP14	1-30

### 2.2 Protein structure prediction

Each hZIP structure was predicted through the state-of-art method for the protein structure prediction AlphaFold2 (Jumper et al., 2021), using as input the mature sequences previously obtained (see Section 2.1). In the case of homodimers predictions, AlphaFold-Multimer v2.2.0 was used (Evans et al., 2022). All models were evaluated accordingly to the per-residue pLDDT score, which corresponds to the model’s predicted score in terms of LDDT-Cα (Mariani et al., 2013). Regions with pLDDT >90 are expected to be modelled with high accuracy. Regions with 70 < pLDDT <90 are good, generally more reliable at backbone level.

Recently, del Alamo and others demonstrated that the AlphaFold2 algorithm can be hacked to sample different conformations of membrane transporters by subsampling the MSA (del Alamo et al., 2022). In particular, varying the depth of the input MSA allows to predict different conformations of transporters and receptors. In this regard, the ColabFold implementation *AlphaFold2_advanced* (https://colab.research.google.com/github/sokrypton/ColabFold/blob/main/beta/AlphaFold2_advanced.ipynb) was used to sample the conformational space of four representative human ZIP proteins (hZIP3, hZIP4, hZIP9 and hZIP11, one for each subgroup) (Mirdita et al., 2022). The settings used were: max_msa_clusters = 32, which determines the number of randomly chosen sequence clusters provided to the AlphaFold2 neural network; and max_extra_msa = 64, which determines the number of extra sequences used to compute additional summary statistics. It must be noted that optimal values of these parameters depend on the particular target protein (del Alamo et al., 2022). Therefore, the minimum values available in the *AlphaFold2_advanced* notebook were used, resulting to be effective in modelling different conformations of the ZIP transporters. The number of random seeds to try was set to 8 to expand the number of obtained models to 40. Finally, the number of recycles was set to one and the minimization option deactivated. Again, the mature sequences of the four hZIPs were used as input, although the hZIP4 was modelled without the ECD (first 300 residues were removed) to reduce the complexity of the prediction.

In order to identify the two three-dimensional models representing the extreme conformations, PCA analyses were performed on the α-carbons resulting 40 models through *pytraj* (Nguyen et al., 2016), a Python package binding to cpptraj program (Roe and Cheatham, 2013). The protein larger loops (e.g., IL2) were excluded from the analyses. In detail, the region excluded were: 109-166 in hZIP3; 1-300 and 402-468 in hZIP4; 79-99 and 268-284 in hZIP9; 102-187 in hZIP11. In the case of hZIP4, given the co-evolutional signal deriving from the homodimerization, two different topologies (see Section 3.2) were found among the 40 structural models. For consistency, only the three-dimensional models with the same topology of the BbZIP crystal structure were included in the PCA analysis.

The conformations at the extremes of the first principal component were extracted. To gain insight into the hypothetical conformational change mechanism, the morphing procedure was applied to the two identified conformations using ChimeraX (Pettersen et al., 2021).

### 2.3 Residues protonation, mutagenesis, and pKa prediction

In the case of hZIP2 and hZIP4, wild-type and mutants, the residues protonation and pKa were predicted using PDB2PQR v3.5.2, with the default PARSE force field, and the implemented PROPKA (Dolinsky et al., 2007; Olsson et al., 2011). The hZIP4 *in silico* double mutants were produced through the SCWRL4 algorithm based on graph theory, which employs an energy function to determine the best conformations for the side-chains (Krivov et al., 2009).

## 3 Results and discussion

### 3.1 Signal peptide

Most of the hZIPs are located on the plasma membrane with the C- and N-terminal ends facing the extracellular space (Guerinot, 2000). hZIP7 and hZIP13 are two exceptions since they are both located on the ER membrane, with hZIP13 also expressed on the Golgi membrane (Lee and Bin, 2019). The C- and N-terminal ends are directed toward the lumen in both cases. Their translocation is often determined by signal sequences that are then removed by post-translational modifications (Zhang et al., 2016). Therefore, the presence of the signal peptide was predicted with the SignalP6.0 web-server (https://services.healthtech.dtu.dk/service.php?SignalP-6.0; Teufel et al., 2022). The results obtained, shown in Table 1, suggest that all members of the LIV-1 subfamily do have a signal peptide of variable length, while members of the other subfamilies (I, II, and GufA) do not. From now on, residue numbers refer to the mature protein sequence.

### 3.2 Extracellular domain

Only the members of the LIV-1 subfamily display an ECD, although with some differences. In particular, members of the subgroup I are characterized by an ECD composed of two independent subdomains: the PCD and the HRD (Zhang et al., 2016). Conversely, members of the subgroups II and III maintain only the PCD, while members of the subgroup IV lack the entire ECD.

Given the high importance in the structure and function of these transporters and the involvement of the ECD in the dimerization (Zhang et al., 2016), three-dimensional models of hZIP4, as a representative member of the LIV-I subfamily, were generated using AlphaFold2 (Evans et al., 2022).

**FIGURE 1:**
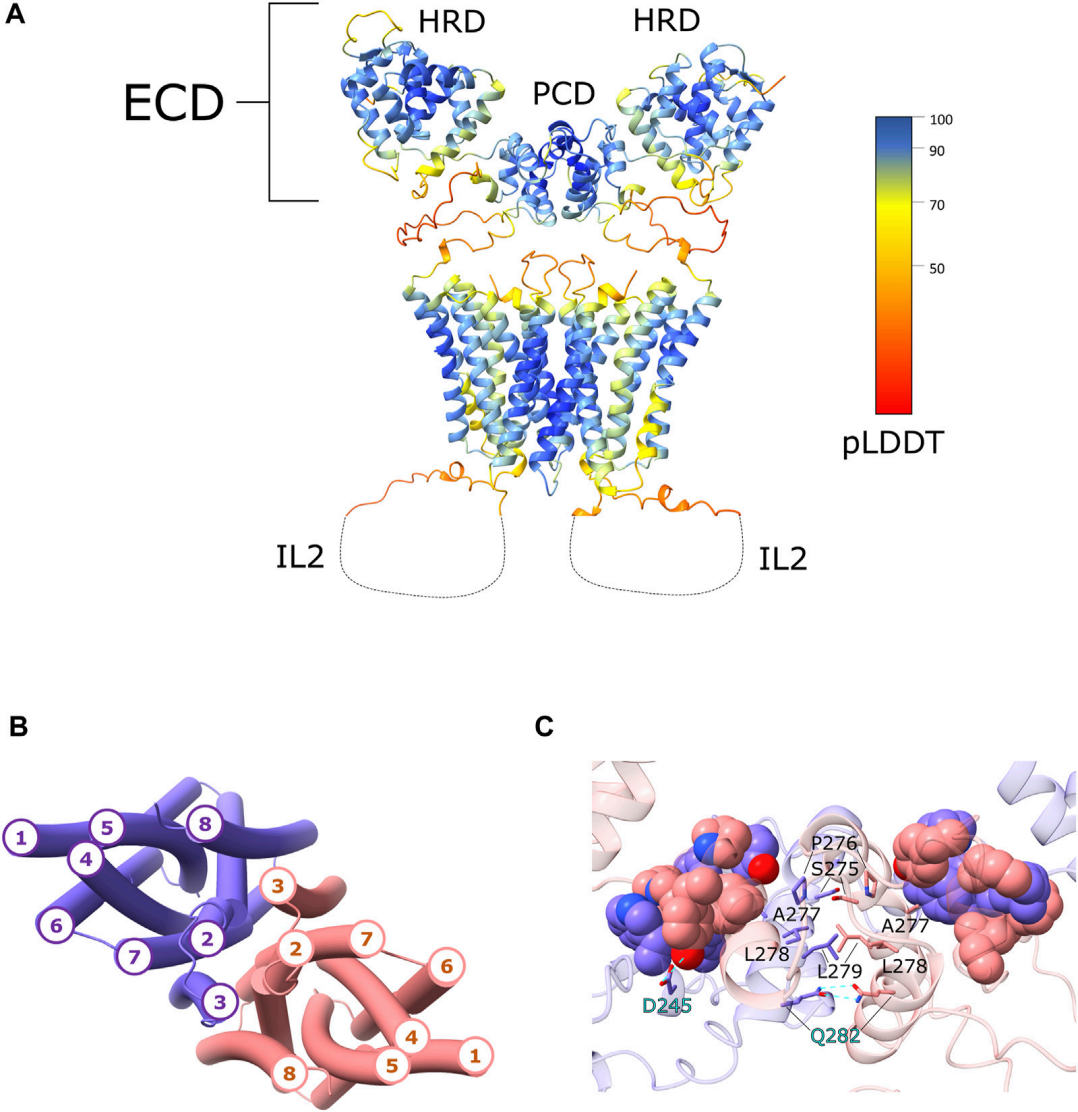
hZIP4 homodimer structural model. **(A)** The three-dimensional model of the hZIP4 homodimer, obtained with AlphaFold-Multimer v2.2.0, is depicted with ribbon representation and colored by pLDDT score. For clarity the long IL2 loop was removed and represented with a dashed line. **(B)** The topology of the eight transmembrane α-helices is here presented. The two chains are colored in purple (chain A) and pink (chain B), in order to distinguish the two monomers. **(C)** Close up on the PCD domain, where the interacting residue of the PAL motifs were represented as sticks. The residues involved in hydrogen bond interactions are represented as sticks and highlighted with cyan labels. The hydrophobic residues forming the hydrophobic core between the two monomers are represented as sphere and colored in purple (chain A) and in pink (chain B).

The interface between the two monomers is predicted to be formed by domain-swapping of the two PCD domains. The serine (S275), proline (P276) and the two leucine (L278 and L279) of the PAL conserved motif, in both monomers, are located at the cross of the two α14s which are at the center of the PCD dimer, similarly to what was observed in the crystal structure of the ZIP4-ECD from *Pteropus alecto* (Zhang et al., 2016). On the contrary, A277 is oriented toward a cluster of hydrophobic residues located on the loop connecting α14 to α13 and on the α10 and H-P linker of the other hZIP4 monomer. Moreover, Q282 (α14), Y182 (α10) and D245 (α12) form hydrogen bonds (Figure 1).

The two monomers are predicted to interact also through the transmembrane α-helices. AlphaFold2 predicted two similar topologies, which differ only for the position of TM3. In one case TM3 was predicted to be in contact with TM2 and TM8 of the same monomer and TM7 of the other monomer, while in the second case TM3 was predicted to be in contact with TM2 and TM7 of the same monomer and TM8 of the other (Figure 1). In both cases TM3 is always in contact with TM2, TM7, and TM8, and this could generate a co-evolutional signal that is misinterpreted by AlphaFold2. Anyhow, comparing the hZIP4 dimer structural models with the BbZIP crystal structure, the second topology with TM3 in contact with TM2 and TM7, was chosen as the correct one. The transmembrane interface of the dimer is predicted to be composed by TM2, TM3, TM7, and TM8. The interacting residues are mostly hydrophobic or aromatic. Noteworthy, the methyl groups of C581, on the two TM7 helices, are predicted to be in contact, although the side-chain rotamers are oriented in opposite directions. It cannot be excluded the formation of a disulphide bond stabilizing the dimer.

### 3.3 Predicted conformational transition

Recently, del Alamo and others demonstrated that the AlphaFold2 algorithm can be exploited to sample different conformations of membrane transporters by subsampling the MSA (del Alamo et al., 2022). In this regard, we used the recent ColabFold implementation *AlphaFold2_advanced*, to sample the conformational space of four representative human ZIP proteins (one for each subgroup).

**FIGURE 2:**
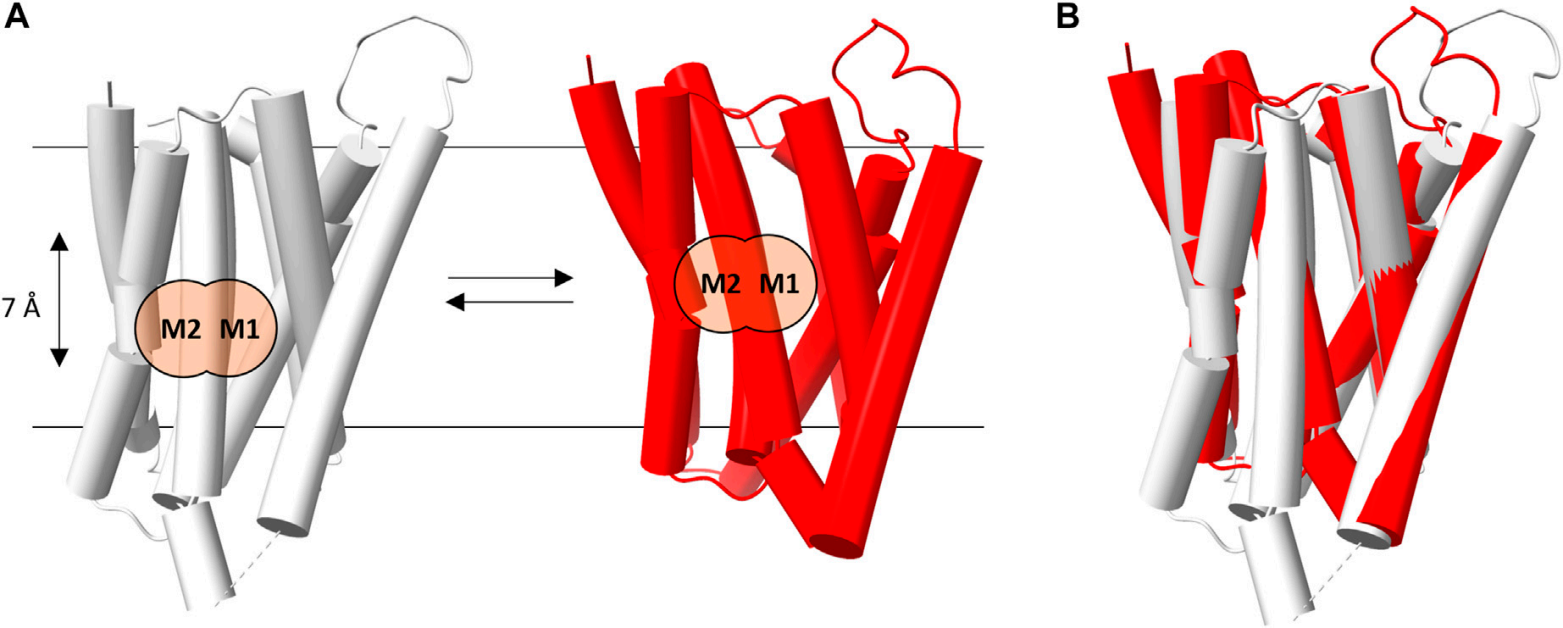
hZIP4 conformational transition. **(A)** Schematic representation of the transition between the inward- (gray) and outward- (red) facing conformations of hZIP4. The M2 and M1 binding sites are marked with black labels and two orange circles, in order two better show the relative position (7 Å apart) of the bimetal cluster in each conformational state. **(B)** Superimposition of the two hZIP4 conformations.

For each of the hZIP modeled, the PCA analysis was applied to identify the two outermost conformations: the first one representing the inward-facing state and the second one representing the outward-facing state (Figure 2 and Supplementary Figures S2, S3).

The morphing procedure, applied between the two conformations, suggests an elevator-type mechanism of transport (see Supplementary Video S1). Indeed, during the predicted conformational transition, the protein appears to be clearly divided into two domains, which is the one that mostly contributes to the conformational transition, and. The present results indicate that the two conformations are characterized by a relative movement of one protein domain (the N-terminal domain composed of TM1, TM4, TM5, and TM6) with respect to the other (the C-terminal domain, composed of TM2, TM3, TM7, and TM8) along an axis perpendicular to the membrane plane, leading to a corresponding movement of the metal binding sites. For instance in hZIP4 the conformational change induces a displacement of the M1 and M2 binding sites of approximately 7 Å across the lipid bilayer (Figure 2). In this case, TM1, TM4, TM5, and TM6 would act as the transport domain, while TM2, TM3, TM7, and TM8 as the scaffold domain. The above described conformational transition perfectly fits the definition of elevator-type transport mechanism (Garaeva and Slotboom, 2020).

### 3.4 Extracellular gating

In the crystallographic structure of BbZIP (PDB ID: 5TSA; Zhang et al., 2017) the transporter is captured in an inward-facing conformation, in which metal ion entry is prevented by a series of hydrophobic residues: a methionine (M99) and an alanine (A102) on TM2, a leucine (L200) and an isoleucine (I204) on TM5, and a methionine (M269) on TM7 (Zhang et al., 2017; Zhang et al., 2020b). From the MSA is evident that these residues are conserved as hydrophobic ones in subfamilies I, II, and GufA, while are substituted with charged or polar amino acids in the subfamily LIV-1 (see Supplementary Materials Fig. 4 and Table 2).

**TABLE 2:** Extracellular hydrophobic plug.

	Res1	Res2	Res3	Res4	Res5
**BbZIP**	M99	A102	L200	I204	M269
**hZIP3**	F55	T58	L200	V204	F273
**hZIP1**	F80	T83	L210	L214	F283
**hZIP2**	F56	A59	L195	V199	F269
**hZIP11**	M51	A54	L233	I237	M302
**hZIP9**	L46	T49	V178	I182	F254
**hZIP7**	L154	D157	T325	V329	F401
**hZIP13**	L85	N88	T218	I222	F296
**hZIP12**	L389	D392	T550	I554	F618
**hZIP4**	V350	D353	T507	V511	F575
**hZIP8**	L151	N154	T314	I318	F381
**hZIP14**	L168	N171	T339	I343	F406
**hZIP6**	L341	D344	T600	V604	F669
**hZIP5**	L233	D236	T392	V396	F461
**hZIP10**	M426	D429	T677	V681	F746

All these residues have been observed to interact in all the predicted outward-facing conformations, forming an hydrophobic plug in the extracellular portion of the protein, hereafter referred to as extracellular hydrophobic plug.

In the BbZIP crystallographic structure, the extracellular hydrophobic plug separates S106 on TM2, from the residues of the BMC on TM4 and TM5. S106 has been observed to interact with the zinc ion by Sharma and Merz during MD simulations, and it has been suggested to be the first extracellular gating residue (Sharma and Merz, 2021). This residue is conserved in hZIP11, while it is substituted by a histidine residue in almost all the hZIP members, apart from hZIP3 (A62), hZIP1 (D87), hZIP9 (V53), hZIP8 (Q158) and hZIP14 (Q175). By simulating the system in the presence of zinc ions, residues directly involved in binding were observed. The pathway of the metal ion toward the mononuclear core was divided into six sequential steps, in which the zinc ion is coordinated by S106, then A102, P199, A203 and finally L200 (Sharma and Merz, 2021). A102 and L200 also correspond to the extracellular hydrophobic plug, suggesting an overlapping mechanism between the substrate first binding and the conformational change. The lack of gating residues conservation within the ZIP family can be explained by the fact that the majority of the metal-protein interactions involve the residues backbone carbonyl and not their side-chain (Table 3).

**TABLE 3:** Extracellular gating residues.

	Res1	Res2	Res3	Res4	Res5
**BbZIP**	A102	S106	P199	L200	A203
**hZIP3**	T58	A62	S199	L200	G203
**hZIP1**	T83	D87	E209	L210	A213
**hZIP2**	A59	H63	Q194	L195	A198
**hZIP11**	A54	S58	N232	L233	G236
**hZIP9**	T49	V53	I177	V178	A181
**hZIP7**	D157	H161	L324	T325	T328
**hZIP13**	N88	H92	L217	T218	A221
**hZIP12**	D392	H396	T549	T550	A553
**hZIP4**	D353	H357	A506	T507	A510
**hZIP8**	N154	Q158	S313	T314	A317
**hZIP14**	N171	Q175	S338	T339	A342
**hZIP6**	D344	H348	S599	T600	A603
**hZIP5**	D236	H240	S391	T392	A395
**hZIP10**	D429	H433	S676	T677	A680

**FIGURE 3:**
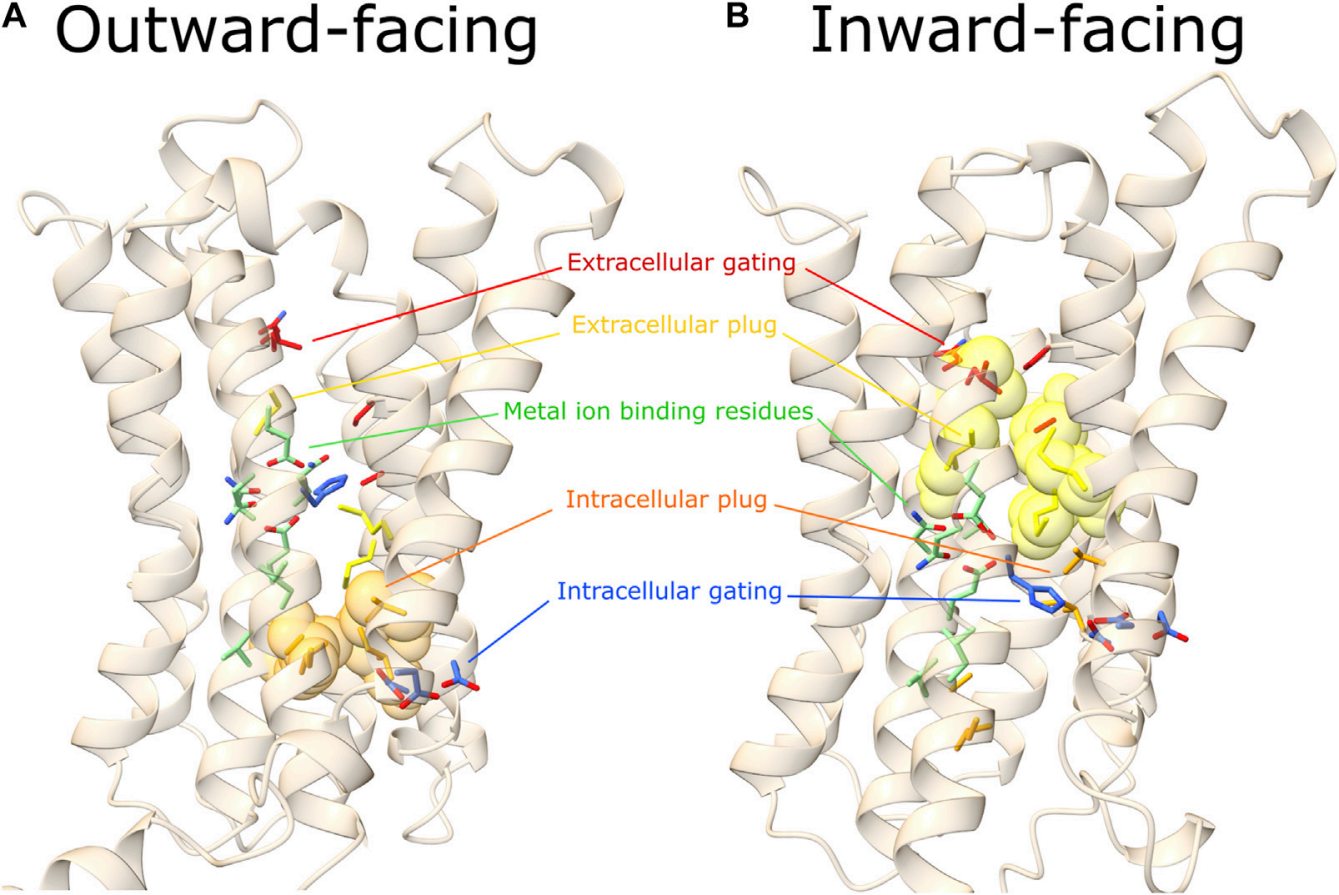
Outward- and inward-facing predicted conformations. Here are represented the outward **(A)** and inward **(B)** predicted conformations of a representative hZIP, hZIP11. In red the extracellular gating residues, in yellow the extracellular hydrophobic plug, in green residues of the TM4 and TM5 extended motifs (involved in metal ion coordination), in orange the intracellular hydrophobic plug, in blue the intracellular gating residues (see Tables 2, 3, 7, 8, 9) for details on hZIP11 residues building up each of the functional motifs indicated). Extracellular and intracellular hydrophobic plug residues are represented as transparent spheres only in the conformational state where the barrier is present.

The elevator-type movement between the N- and C-domain, during the conformation transition, would be responsible for the disruption of the extracellular hydrophobic plug, allowing the first gating residue and a polar residue on TM5 (P1′-TM5, see Section 3.5), to be within a distance compatible with the coordination of a divalent metal ion (Figure 3). Two exceptions are represented by hZIP9 and hZIP3. In hZIP9, the residue E57, one α-helix turn above V53, is close enough (∼4.2 Å, evaluating other side-chain rotamers) to H185 to be compatible with zinc ion coordination (∼2.1 Å). In hZIP3 case, there is no polar residue on TM2. However, in the outward conformation, a residue on TM6, Q266, approaches H180, P1 in the TM4-motif, with a distance compatible with metal ion coordination (∼4 Å, evaluating side-chain rotamers).

In the predicted outward conformations, an additional core of hydrophobic residues in the cytoplasmic region (hereafter referred to as intracellular hydrophobic plug) prevents the leak of substrate towards the cytosol, blocking the path between the M1 binding site and the intracellular gating residues (see Section 3.6).

### 3.5 Transmembrane metal binding site

**FIGURE 4:**
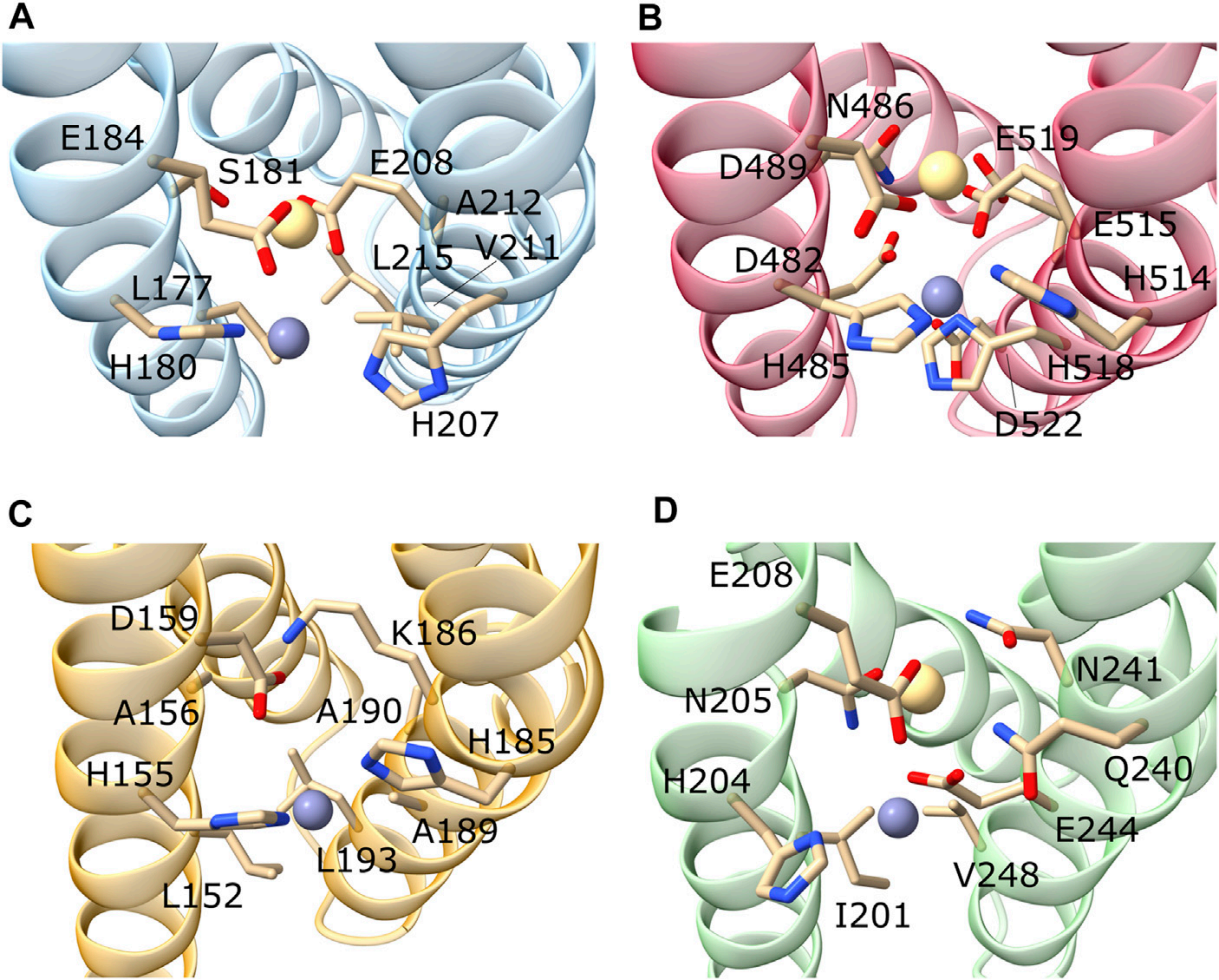
Focus on the M1 and M2 binding sites of representative hZIP members. Here are represented the predicted M1 and M2 binding sites of hZIP3 **(A)**; hZIP4 **(B)**; hZIP9 **(C)**; hZIP11 **(D)**. The metal ions were inserted by superimposing the models onto the BbZIP crystal structure. In the case of hZIP9, the metal in M2 was removed as K186 blocks the access to the metal binding site.

From the BbZIP crystal structure was evident the presence of two transmembrane metal-binding sites composed by six residues of the two P1-P2-x-x-P3 motifs on TM4 and TM5 where the x is usually a small or hydrophobic amino acid). P1, P2, and P3 are involved in the coordination of the transported metal at the two metal binding sites M1 and M2 (Figure 4) (Zhang et al., 2020b). However, this motif can be further expanded to include other conserved residues, as also suggested by Zhang and others (2020). The TM4 motif can be expanded to P3′-x-x-P1-P2-x-x-P3 to include a fourth residue (P3′) usually an acidic residue as P3, although only conserved in the LIV-1 subfamily members (Supplementary Figure S4).

Similarly, the TM5 motif can be further expanded to include two additional residues becoming P1′-P2′-x-x-P1-P2-x-x-P3. In fact, the P1′ and P2′ residues are usually a histidine and a glutamate, respectively, as are the P1 and P2 residues, respectively (Supplementary Materials Fig. 4). The TM5 motif is strongly conserved among the LIV-1 family members (Table 4).

**TABLE 4:** Subgroup LIV-1 TM4 and TM5 BMC residues.

	TM4				TM5				
	P3′	P1	P2	P3	P1′	P2′	P1	P2	P3
**hZIP4**	D482	H485	N486	D489	H514	E515	H518	E519	D522
**hZIP12**	D525	H528	N529	D532	H557	E558	H561	E562	D565
**hZIP5**	D367	H370	N371	D374	H399	E400	H403	E404	D407
**hZIP6**	D575	H578	N579	D582	H607	E608	H611	E612	D615
**hZIP10**	D652	H655	N656	D659	H684	E685	H688	E689	D692
**hZIP7**	D326	H329	N330	D333	H358	E359	H362	E363	D366
**hZIP13**	D225	D228	N229	H232	H257	E258	H261	E262	D265
**hZIP8**	D289	H292	N293	D296	E321	E322	H325	E326	D329
**hZIP14**	D314	H317	N318	D321	E346	E347	H350	E351	D354

#### 3.5.1 Subfamily I

The only member of the human ZIP subfamily I, hZIP9, displays the conservation of only the TM4-P1 (H155), TM4-P3 (D159) and TM5-P1’ (H185) residues (Table 5). This suggests that the second metal binding site M2 is lacking in this transporter (Figure 4). The residue at the TM5-P2’ (K186) could occupy the M2 binding site mimicking the metal ion, as also suggested for the hZIP2 transporter (Gyimesi et al., 2019). However, differently from hZIP2, hZIP9 has a valine (V53) in the same position of the H63, responsible for the pH-dependent activity in hZIP2 (Gyimesi et al., 2019).

**TABLE 5:** Subgroup I TM4 and TM5 BMC residues.

	TM4				TM5				
	P3′	P1	P2	P3	P1′	P2′	P1	P2	P3
**hZIP9**	L152	H155	A156	D159	H185	K186	A189	A190	L193

In the outward-facing conformation, the M1 residues are surrounded by several hydrophobic amino acids which prevent the release of the metal ion towards the cytosol. In detail, L46, L152, A189, and V257 hinder the path toward H261 and D123, the two highly conserved residues most likely involved in the attraction and release of Zn^2+^ (see Section 3.6). In the inward-facing conformation, the metal binding residues of the M1 site result to be still blocked by the intracellular hydrophobic plug and by the additional M119 and L152. In this regard, an additional conformational transition is needed for the substrate release.

Interestingly, hZIP9 was demonstrated to be a ZIP transporter able to mediate testosterone-induced non-classical signaling coupled with a G-protein, causing increment of intracellular Zn^2+^and inducing apoptosis (Thomas et al., 2018). The interaction between hZIP9 and a G-protein could be the trigger responsible for the opening of the hydrophobic plug allowing the release of the substrate. With this aim, AlphaFold2 was employed to predict a hypothetical complex between hZIP9 and two G-proteins experimentally identified. Two interfaces were predicted by AlphaFold2, one involving the end of TM4 and the shorter IL2 loop, and another formed by the loop connecting TM8 to TM7 (Supplementary Figure S5). Although the interacting regions of the G-proteins are not the canonical ones, the binding interfaces are close to the nucleotide binding site, suggesting that complex formation can affect the nucleotide affinity.

#### 3.5.2 Subfamily II

In the hZIP subfamily II, the TM4-motif is LxxHSxxE, where the presence of a glutamate residue in the place of an aspartate in P3 could compensate for the shorter side-chain of a serine in the place of an asparagine in P2 (Table 6).

**TABLE 6:** Subgroup II TM4 and TM5 BMC residues.

	TM4				TM5				
	P3′	P1	P2	P3	P1′	P2′	P1	P2	P3
**hZIP1**	L187	H190	S191	E194	H217	K218	L221	A222	L225
**hZIP2**	L172	H175	S176	E179	H202	K203	V206	V207	V210
**hZIP3**	L177	H180	S181	E184	H207	E208	V211	A212	L215

The TM5-motif is H[K/E]xx[V/L][A/V]xx [L/V]. The presence of hydrophobic residues in TM4-P1′, TM5-P1,-P2 and -P3, indicates the loss of the second metal binding site M2 (Table 6). This characteristic, as well as the presence of a lysine residue in P2′ position (except for hZIP3), is observed also in hZIP9. While from the hZIP3 structural model (Figure 4), it appears that the M2 is conserved, the absence of a BMC in the other two members of subfamily II was confirmed by studies carried out on the hZIP4 mutants (Zhang et al., 2020b). In particular, the hZIP4-∆M2 mutant was obtained by mutating N486 and E515 to alanine, in order to understand the impact of the absence of M2 (Zhang et al., 2020b). Mutation reduced the Zn^2+^ transport by approximately 30%. The residual activity observed in the hZIP4 mutants indicates that M2, at least *in vitro*, is not essential for metal transport.

Moreover, the hZIP4-∆M2 showed a pH dependent activity, at variance with the wild-type protein. This is also the case for hZIP2, whose pH-dependent activity was analyzed, suggesting H63 to be the residue critical for the pH sensitivity (Gyimesi et al., 2019).

Interestingly, in the hZIP2 model, H63 precedes the M1 residues, and it corresponds to the hypothetical first BbZIP gating residue S106.

It should be noted that H63 is not conserved among the members of the human ZIP subfamily II. In fact, in the same position hZIP1 displays an aspartate residue (D87), while hZIP3 an alanine residue (A62).

#### 3.5.3 Subfamily LIV-1

The subfamily LIV-1 members display a conserved TM4 motif: DxxHN[F/L]xD (Table 4). In this regard, hZIP13 has an asparagine residue in P1 (N193), whereas P2 (D196) and P3 (H200) are inverted with respect to the canonical TM4 motif. Noteworthy, in *drosophila*, the human ZIP13 orthologue has been observed to transport iron ions from the cytosol to the Golgi lumen, an opposite transport direction with respect to the canonical mechanism (Xiao et al., 2014). In this regard, the presence of a histidine in P3 could have a role in the attraction of the cytosolic metal ion, for the inverse transport to the Golgi lumen.

The TM5 motif HExxHExxD is conserved, although the P1′ residue is substituted by a glutamate in both hZIP8 (E321) and hZIP14 (E346). In this regard, this substitution has been suggested to be involved in an increased metal selectivity of these transporters for manganese, as indeed observed experimentally (Fujishiro and Kambe, 2022).

The LIV-1 members maintain the canonical BMC, with the residues included in the expanded motifs (TM4-P3′, TM5-P1′ and TM5-P2′) in the proximity of the metal binding sites. TM4-P3′ could be involved in the release of the substrate from the BMC, while TM5-P1′ and TM5-P2′ could have a role in attracting the metal ions toward the BMC. Indeed, a key role for these residues is supported by experimental data (Antala et al., 2015; see also https://www.ncbi.nlm.nih.gov/clinvar/RCV000348067/).

Here, the structural models of the previously mentioned hZIP4 mutants (hZIP4-∆M2 and hZIP4-mutantK) were generated and analyzed to rationalize the results of biochemical studies. The two models were produced mutating the AlphaFold2 hZIP4 model residues with SCWRL4 (Krivov et al., 2009). In the hZIP4-mutantK E515 was mutated to K515 and N486 to S486. In the corresponding structural model, K515 interacts with D482, making E519 inaccessible. In this case, D482 and E519 would not be able to interact with the metal ion. Conversely, in the hZIP4-∆M2 the two alanine residues do not cause steric hindrance (see Data Availability Statement for Zenodo Repository). This could explain why hZIP4-mutantK activity is significantly reduced with respect to hZIP4-∆M2.

Furthermore, the hZIP4-∆M2 mutant displayed a transport activity dependent on pH. In order to find a possible explanation for this behavior and to compare it to the hZIP2 pH-dependent activity, the pKa of the hZIP4 and hZIP4-∆M2 residues has been predicted with PDB2PQR v3.5.2 and the implemented PROPKA (Dolinsky et al., 2007; Olsson et al., 2011). The pKa prediction did not show significant differences between the mutant and wild type residues. However, H514 was predicted to have a pKa of 9.16 in hZIP4 wild type, reduced to 7.32 in the M2 mutant. Given that this residue is close to H357, corresponding to the hZIP2 H63, and to E515/A515, it is reasonable to think that the double alanine mutant affects the H514 and H357 protonation state at physiological pH.

#### 3.5.4 Subfamily GufA

In humans, the GufA subfamily includes only hZIP11. This transporter is the orthologue of the bacterial BbZIP and displays the patterns IxxHNxxE as TM4-motif, and QNxxEGxxV as TM5-motif (Table 7).

**TABLE 7:** Subgroup GufA TM4 and TM5 BMC residues.

	TM4				TM5				
	P3′	P1	P2	P3	P1′	P2′	P1	P2	P3
**hZIP11**	I201	H204	N205	E208	Q240	N241	E244	G245	V248

To visualize these residues in the context of the three-dimensional structure of the transporters, the structural model of hZIP11 and the crystallographic structure of BbZIP were superimposed (Zhang et al., 2017) (Supplementary Figure S6). As can be seen from the figure, the TM4 and TM5 of hZIP11 perfectly overlap with the corresponding helices of BbZIP, and the residues involved in metal binding at M1 and M2 also display the same orientation (Supplementary Figure S6). Thus, as initially observed by sequence alignment, and later confirmed by structural superimposition, hZIP11 and BbZIP exhibit high structural homology, and presumably also functional homology in terms of specificity, especially at the metal center. Based on these results, the metal center of hZIP11 can be predicted to be binuclear, likewise that described for BbZIP.

#### 3.6 Intracellular gating

Analysis of the BbZIP crystal structure allowed to identify three additional metal binding sites (Zhang et al., 2017). M5 is right under the BMC on the cytoplasmic side, and is formed by H177 (TM4-P1) and E276 (TM7-M5). These two residues are strongly conserved among all the hZIP transporters (E276 is often substituted by an aspartate). The only exceptions are: hZIP13, with an aspartate in TM4-P1 and a glutamine in TM7-M5; hZIP7 present a serine in TM7-M5; hZIP9 an histidine in TM7-M5 (Table 8).

**TABLE 8:** Intracellular gating residues.

	TM4-M5	TM7-M5	TM3-M6	TM3-M3
**BbZIP**	H177	E276	D144	H275
**hZIP3**	H180	E280	E102	L279
**hZIP1**	H190	E290	E127	L289
**hZIP2**	H175	E276	E120	L275
**hZIP11**	H204	D309	D96	D308
**hZIP9**	H155	H261	D123	V260
**hZIP7**	H329	S408	E208	V407
**hZIP13**	D228	N303	E134	V302
**hZIP12**	H528	E625	E439	V624
**hZIP4**	H485	D582	E395	C581
**hZIP8**	H292	D388	E191	A387
**hZIP14**	H317	D413	E208	A412
**hZIP6**	H578	D676	E410	V675
**hZIP5**	H370	D468	E278	V467
**hZIP10**	H655	D753	E487	V752

The last two binding sites observed in BbZIP are M6 and M3, constituted by D144 (TM3-M6; TM3-M3) and H275 (TM7-M3). D144 is strongly conserved (as aspartate or glutamate) among the fourteen members, while H275 is always substituted with a hydrophobic residue, with the exception of hZIP11 in which an aspartate is present (D308).

As mentioned in the previous paragraph (see Section 3.4), in the outward-facing conformation, the intracellular hydrophobic plug blocks the release of the metal ion towards the intracellular gating residues, which then release it into the cytosol. The intracellular plug residues (ɸ_x_ being a hydrophobic residue) are mainly conserved in four positions (Table 9). In detail, the first residue is located three positions above the TM4-P3’ (ɸ_1_-x-x-x-P3′), the second is found two residues after TM5-P3 (P3-x-x-ɸ_2_), and the third and fourth are respectively three residues before (ɸ_3_-x-x-x-M5) and right after the TM7-M5 intracellular gating residue (M5-ɸ_4_). The only exceptions are hZIP7, hZIP13 and hZIP9, in which ɸ_1_ is substituted by an asparagine (N296, hZIP7; N189, hZIP13) or a threonine (T148, hZIP9). Moreover, in the outward-facing conformation structural model of hZIP11, A247 appears to participate in the hydrophobic plug in the place of P251.

**TABLE 9:** Intracellular hydrophobic plug.

	Res1	Res2	Res3	Res4
**BbZIP**	F170	A218	V272	V277
**hZIP3**	L173	S218	I276	I281
**hZIP1**	L183	R228	I286	I291
**hZIP2**	L168	R213	V272	I277
**hZIP11**	L197	A247	V305	I310
**hZIP9**	T148	F196	V257	V262
**hZIP7**	N296	I343	V404	V409
**hZIP13**	N189	I236	I299	V304
**hZIP12**	I521	V568	L621	M626
**hZIP4**	I478	A525	V578	M583
**hZIP8**	I285	I332	I384	M389
**hZIP14**	I310	I357	I409	M414
**hZIP6**	V571	V618	V672	M677
**hZIP5**	V363	M410	V464	M469
**hZIP10**	V648	V695	V749	M754

The conformational transition from the outward-to the inward-facing state disrupts the intracellular hydrophobic plug, while the formation of the extracellular hydrophobic plug prevents the bound metal ions to diffuse backwards, facilitating the interactions with the intracellular gating residues on TM4 and TM7.

In this regard, the intracellular loop IL2 has been demonstrated to play a role in binding zinc ions in the cytosol, regulating post-translational modifications of the loop that promote internalization (Zhang et al., 2020a). It should be noted that in hZIP9 IL2 is significantly shorter, suggesting a different regulation mechanism.

## 4 Conclusion

In this manuscript we have described a comprehensive *in silico* structural analysis of the human ZIP family members that sheds light on the molecular details of metal binding and translocations path. Indeed, one of the most important results of this analysis has been obtained by using a recent implementation of the AlphaFold2 algorithm coupled to subsampling of the multiple sequence alignment. This allowed to obtain models of the transporters in two different conformations, corresponding to the outward-facing and inward-facing states. Comparison of the two conformational states allowed to hypothesize an “elevator-type” mechanism for ion translocation. In particular, it is evident from the models that the relative movement of the two protein domains causes the formation of a hydrophobic plug on the extracellular side (or the intraluminal side for intracellular transporters) and the disruption of a hydrophobic plug on the intracellular side, allowing metal release. Further, the relative movement of the two domains allows TM4-P1 residue to drag the metal from the BMC to the intracellular gating residues. The movement of the substrate-binding site across the membrane, and the presence of two “barriers” and two “gates” that form upon the conformational transition, indicates that the ZIP protein family members could act through moving barriers with two gates, as envisioned in the comprehensive work of Garaeva and Slotboom (2020) for the elevator-type mechanism taking place in bile acid transporter ASBT. During the revision of the present work, we became aware of a recently published manuscript describing an elevator-type mechanism for BbZIP (Wiuf et al., 2022). Thus, the transport mechanism here hypothesized is fully supported by the study of Wiuf et al. (2022). In conclusion, the availability of structural models for all members of the hZIP family of metal transporters will be a valuable aid in the experimental characterization of the functional details of metal transport.

## Data Availability

The original contributions presented in the study are included in the article/Supplementary Material, the data generated and analyzed for this study can be found in the Zenodo repository: 10.5281/zenodo.6900558.
