# Supplementary material for: A computational study of the structure and function of human Zrt and Irt-like proteins metal transporters: An elevator-type transport mechanism predicted by AlphaFold2
Source: Front Chem. 2022 Sep 20;10:1004815. doi: 10.3389/fchem.2022.1004815 (PMC9530640; doi:10.3389/fchem.2022.1004815)
Supplement: Supplementary file 2 [file DataSheet1.docx]

Supplementary Material

A computational study of the structure and function of human ZIP metal transporters: an elevator-type transport mechanism predicted by AlphaFold2

Pasquadibisceglie Andrea^1^, Leccese Adriana^1^, Polticelli Fabio^1,2*^

^1^Department of Sciences, Roma Tre University, 00146 Rome, Italy

^2^National Institute of Nuclear Physics, Roma Tre Section, 00146 Rome, Italy

*** Correspondence:**

Fabio Polticelli

Department of Sciences, Roma Tre University

Viale Guglielmo Marconi 446, 00146 Rome, Italy

Tel. +39-06-57336362

Fax. +39-06-57336321

e-mail [fabio.polticelli@uniroma3.it](mailto:fabio.polticelli@uniroma3.it)

**Supplementary Table 1. hZIP common names and corresponding UniProt ID**

| **Common name** | **UniProt ID** |
| --- | --- |
| hZIP1 | Q9NY26 |
| hZIP2 | Q9NP94 |
| hZIP3 | Q9BRY0 |
| hZIP4 | Q6P5W5 |
| hZIP5 | Q6ZMH5 |
| hZIP6 | Q13433 |
| hZIP7 | Q92504 |
| hZIP8 | Q9C0K1 |
| hZIP9 | Q9NUM3 |
| hZIP10 | Q9ULF5 |
| hZIP11 | Q8N1S5 |
| hZIP12 | Q504Y0 |
| hZIP13 | Q96H72 |
| hZIP14 | Q15043 |


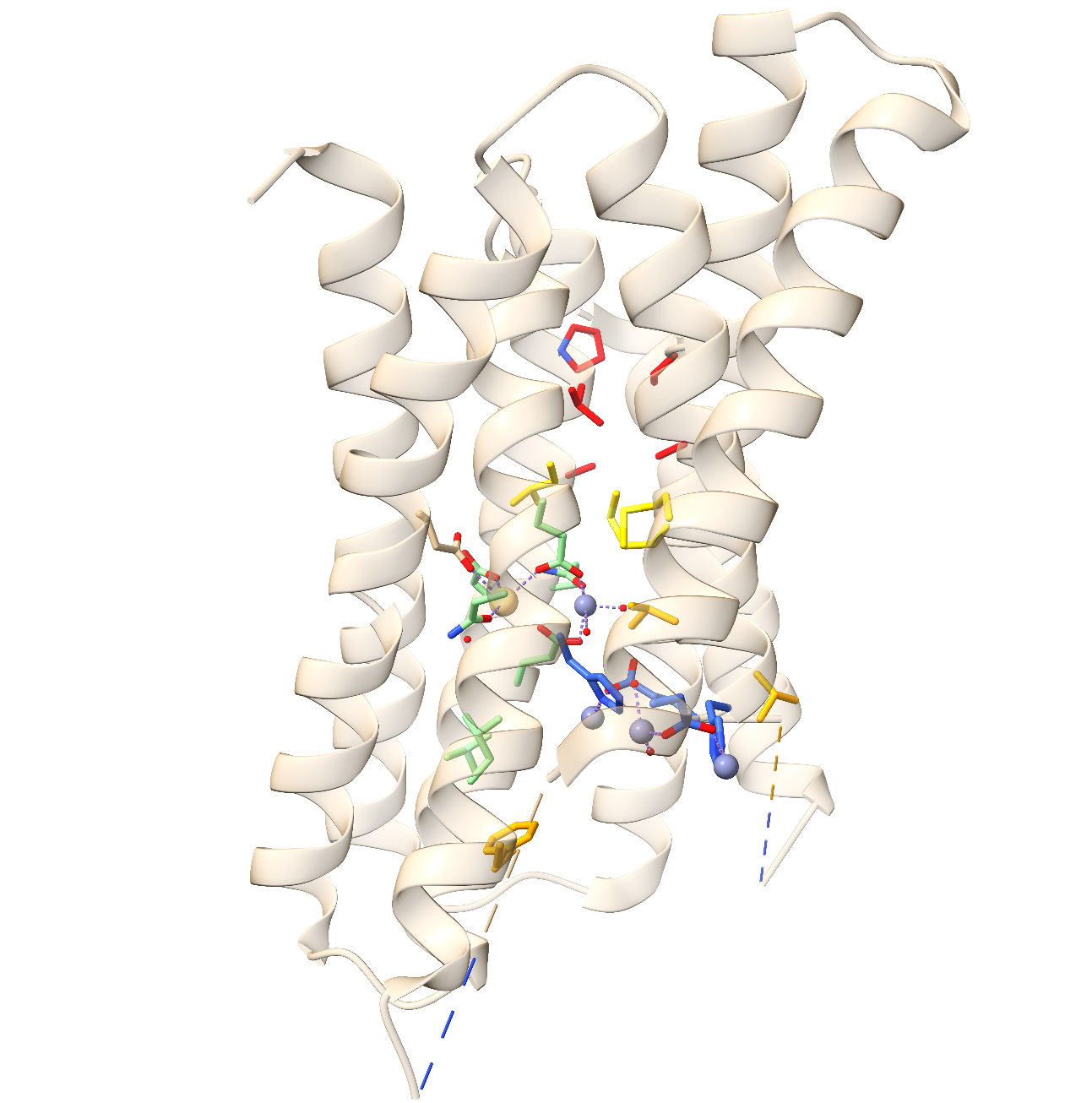


**Supplementary Figure 1. Schematic representation of the BbZIP crystal structure.** The protein backbone is represented as ribbons, while residues as sticks. The extracellular gating residues are colored in red; the extracellular hydrophobic plug residues in yellow; the TM-4 and TM5 extended motifs residues in light green; the intracellular hydrophobic plug residues in orange; the intracellular gating residues in blue.


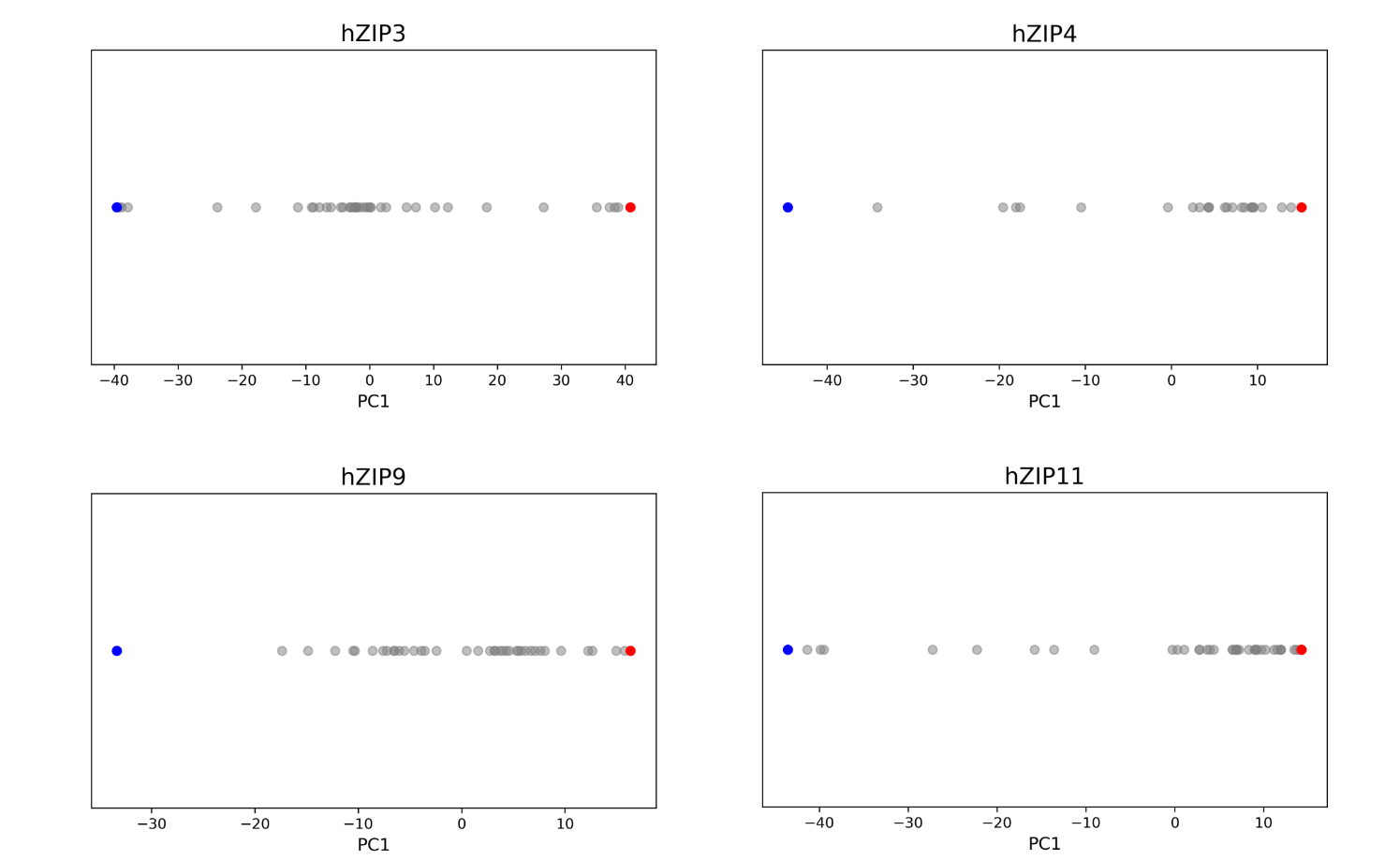


**Supplementary Figure 2. Plot of the first component extracted from the PCA analyses performed on the four hZIPs.** The three-dimensional models at the extremes of the first component are highlighted in red and in blue.

**
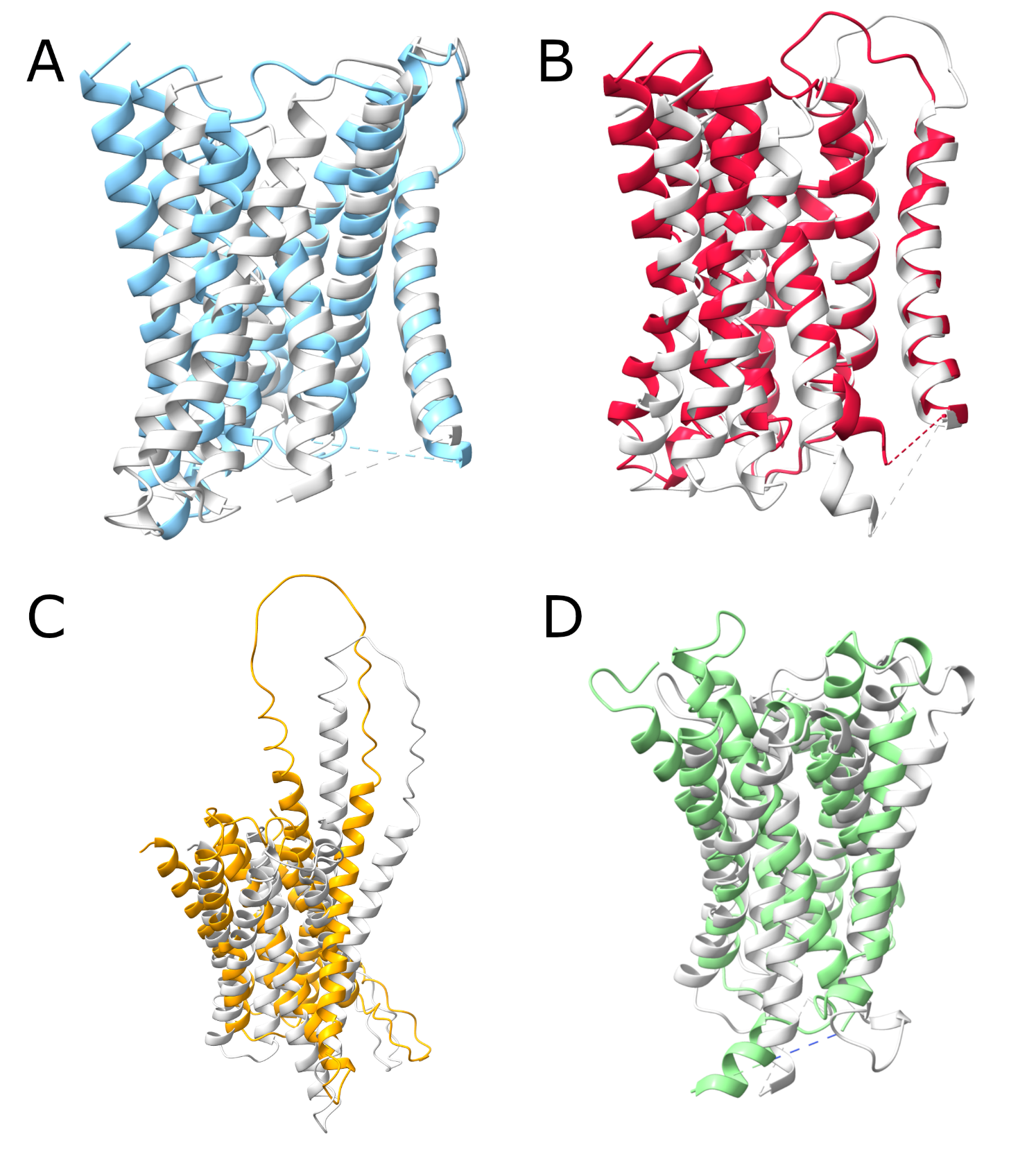
**

**Supplementary Figure 3. Superimposition of the identified conformations for each hZIP.** The inward-facing conformations are colored in grey, while the outward is colored in blue for hZIP3 (A), in red for hZIP4 (B), in orange for hZIP9 (C), in green for hZIP11 (D).


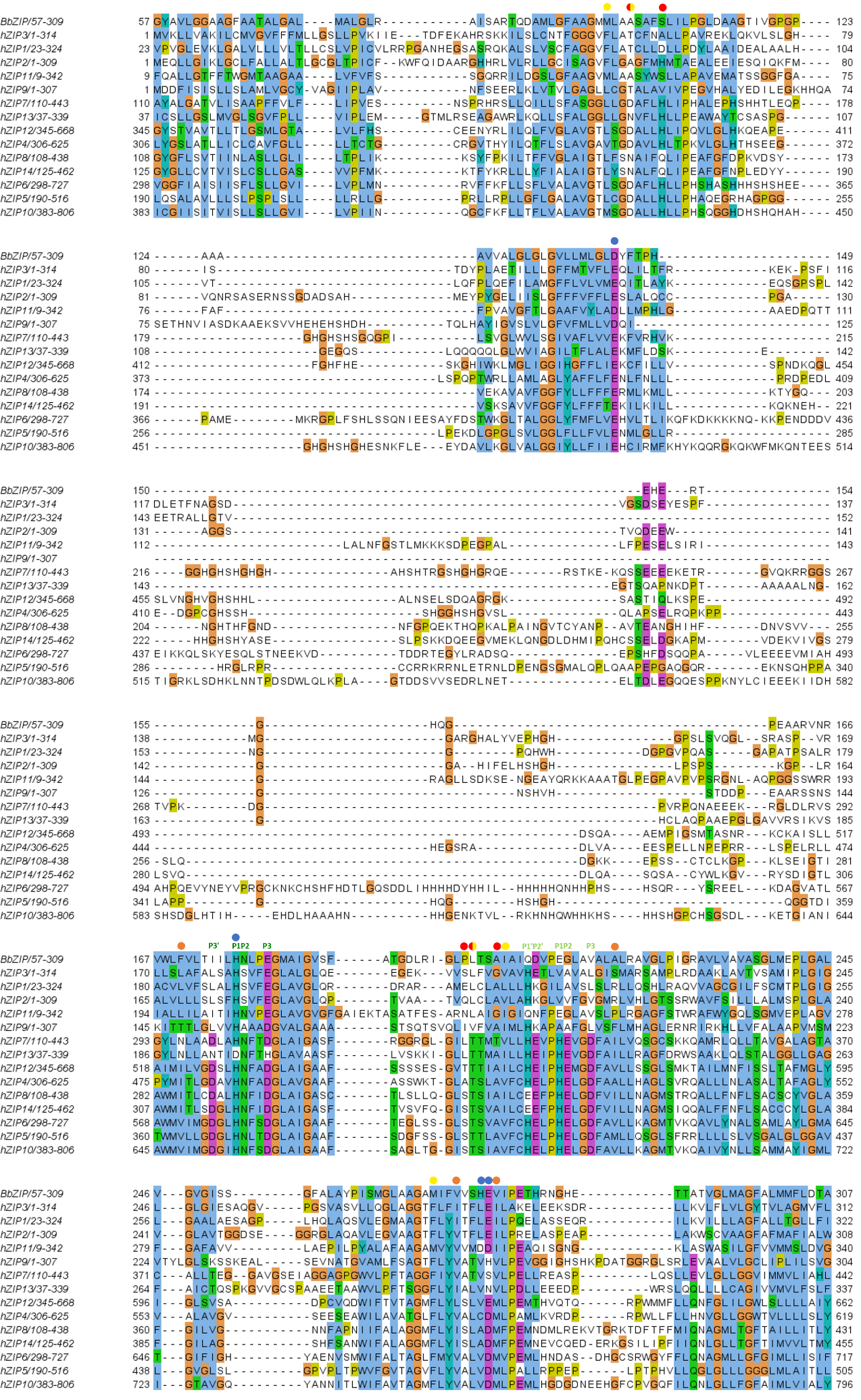


**Supplementary Figure 4. Multiple sequence alignment.** Sequences were trimmed to include only the 8 transmembrane α-helices and the loops connecting them. The important residues have been highlighted as follows: the extracellular hydrophobic plug residues with yellow circles; the extracellular gating residues with red circles; the TM4-extended motif residues with dark green characters; the TM5-extended motif residues with light green characters; the intracellular hydrophobic plug with orange circles; the intracellular gating residues with blue circles.


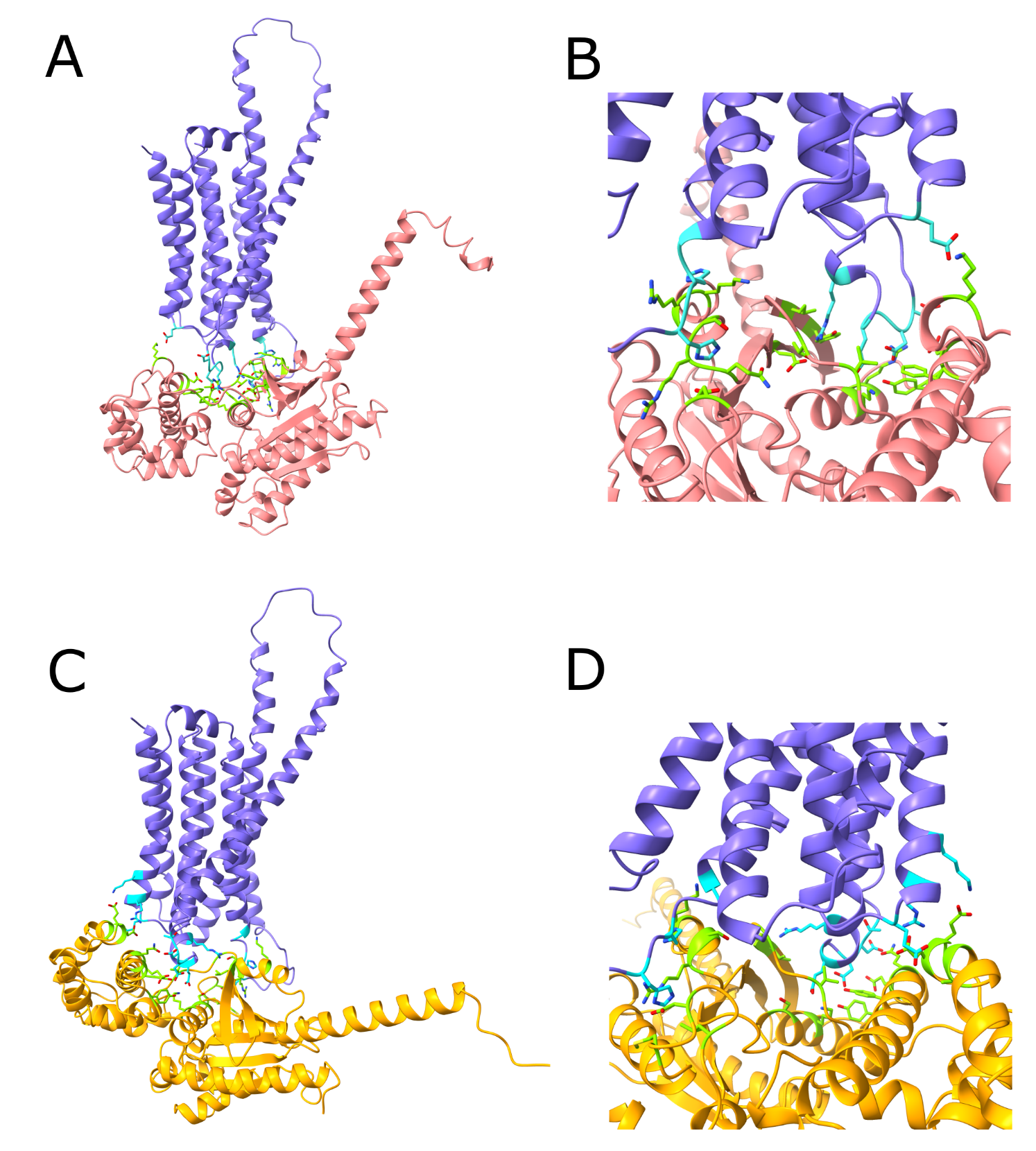


**Supplementary Figure 5. hZIP9-G protein predicted complexes.** Here are reported the two macromolecular complexes between hZIP9 (in purple) and the G-protein Gnα11 (in pink) (UniProt ID: P29992) (A, B) and between hZIP9 and the G-protein Gnαi1 (in orange) (UniProt ID: P63096) (C, D). Interacting residues are shown as stick and colored in cyan (hZIP9) and in green (G-proteins).


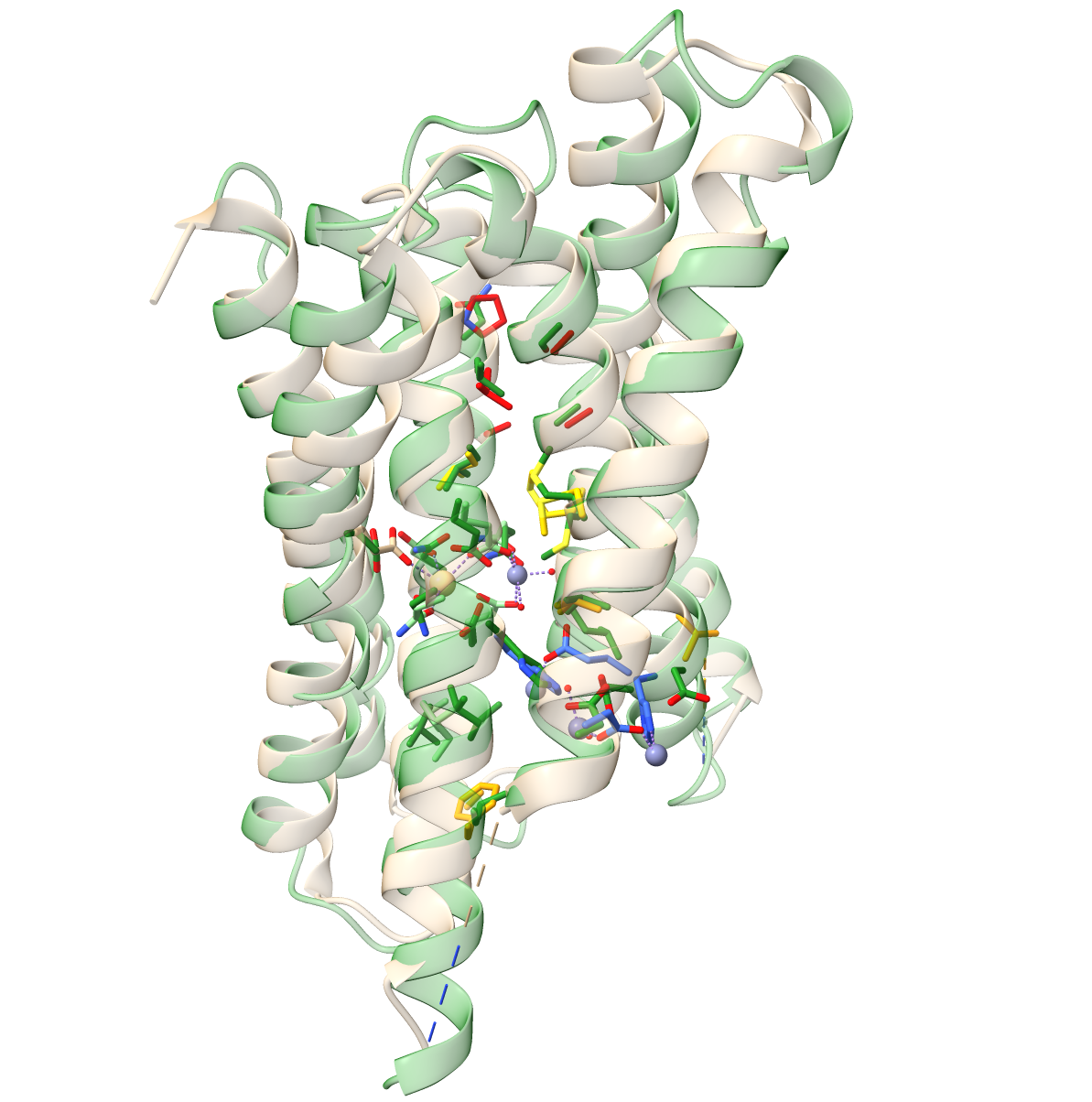


**Supplementary Figure 6. Superimposition between the BbZIP crystal structure and the hZIP11 structural model.** The BbZIP crystal structure (in tan) and the hZIP11 structural model (in green) have been superimposed. The critical residues are represented as stick. For the BbZIP the extracellular gating residues are colored in red; the extracellular hydrophobic plug residues in yellow; residues of the TM4 and TM5 extended motifs in light green; the intracellular hydrophobic plug residues in orange; the intracellular gating residues in blue. In the case of the hZIP11, all the residues have been colored in dark green.
